# 3D Printing for Bone-Cartilage Interface Regeneration

**DOI:** 10.3389/fbioe.2022.828921

**Published:** 2022-02-14

**Authors:** Jialian Xu, Jindou Ji, Juyang Jiao, Liangjun Zheng, Qimin Hong, Haozheng Tang, Shutao Zhang, Xinhua Qu, Bing Yue

**Affiliations:** ^1^ Department of Bone and Joint Surgery, Renji Hospital, School of Medicine, Shanghai Jiao Tong University, Shanghai, China; ^2^ The First Clinical Medical College, Shandong University of Traditional Chinese Medicine, Jinan, China

**Keywords:** 3D printing, bone repair, chondral regeneration, reconstructive implant, regenerative medicine

## Abstract

Due to the vasculature defects and/or the avascular nature of cartilage, as well as the complex gradients for bone-cartilage interface regeneration and the layered zonal architecture, self-repair of cartilage and subchondral bone is challenging. Currently, the primary osteochondral defect treatment strategies, including artificial joint replacement and autologous and allogeneic bone graft, are limited by their ability to simply repair, rather than induce regeneration of tissues. Meanwhile, over the past two decades, three-dimension (3D) printing technology has achieved admirable advancements in bone and cartilage reconstruction, providing a new strategy for restoring joint function. The advantages of 3D printing hybrid materials include rapid and accurate molding, as well as personalized therapy. However, certain challenges also exist. For instance, 3D printing technology for osteochondral reconstruction must simulate the histological structure of cartilage and subchondral bone, thus, it is necessary to determine the optimal bioink concentrations to maintain mechanical strength and cell viability, while also identifying biomaterials with dual bioactivities capable of simultaneously regenerating cartilage. The study showed that the regeneration of bone-cartilage interface is crucial for the repair of osteochondral defect. In this review, we focus on the significant progress and application of 3D printing technology for bone-cartilage interface regeneration, while also expounding the potential prospects for 3D printing technology and highlighting some of the most significant challenges currently facing this field.

## 1 Introduction

During activities such as walking, kneeling, rotating and jumping, the knee joint is subjected to compression, shear and tension forces from the whole body, where the bone-chondral interface serves as a transitional interface between viscoelastic cartilage and solid bone, maintaining structural stability (Hoemann et al., 2012). Osteochondral damage (OCD) disrupts the integrity and stability of the bone-cartilage interface as it includes not only the articular cartilage but also the underlying subchondral bone (Madry et al., 2010). OCD is often caused by trauma, cancer, and joint inflammation, such as osteoarthritis (OA) (Mano and Reis, 2007). As we age, the natural wear and tear of cartilage tissue often leads to OA, which can evolve into OCD and is difficult to effectively treat, with many patients continuing to suffer from pain that can impede even simple daily tasks, such as walking, and can progress to physical disability (Glyn-Jones et al., 2015; Sacitharan, 2019). OA affects 7% of the global population (∼500 million people) and is significantly more common in women than men (Mandl, 2019; Hunter et al., 2020).

Damage or degeneration at the bone-cartilage interface due to osteochondral defects is difficult to self-heal and often requires external therapies due to the complex structural features of the osteochondral structure. Common surgical treatments for OCD occurring in large areas currently used in clinical practice include autologous chondrocyte implantation (ACI) (Kubosch et al., 2018; Schuette et al., 2021), osteochondral allograft transplantation (OCA) (Gilat et al., 2021) and matrix-induced autologous chondrocyte implantation (MACI) (Gao et al., 2019). Each of these treatment strategies has demonstrated a certain level of success. Autologous bone has good ability to induce osteogenesis and integrate with defect area, but there is a scarcity of available OCA donors (Moatshe and LaPrade, 2020), and OCA surgery will have adverse effects on the donor-site (Hishimura et al., 2019). ACI and MACI have valuable advantages such as good biocompatibility, small trauma, quick recovery after operation and reconstruction of tissue function. However, ACI or MACI involves two separate surgeries, which increases a greater risk of severe graft site infections that has been reported by Gobbi et al. (2020). Therefore, an eminent push toward the development of new treatment options that possess better treatment effects and less disadvantages. High expectations are given to the 3D bioprint technology.

In recent years, 3D bioprinting, an additive manufacturing technology, has reformed the field of regenerative medicine and tissue engineering (TE). To date, 3D printing technology has been implemented in anatomical tissue models, medical devices, elucidation of biological mechanisms, TE scaffolds, and drug delivery routes (Murphy and Atala, 2014; Ventola, 2014; Schweiger et al., 2016; Liaw and Guvendiren, 2017). 3D bioprinting is an integrated process that requests consideration of different design factors, including imaging (CT or MRI), modeling (computer-aided design (CAD), computer-aided manufacturing tools, and mathematical modeling), printer selection, bioink selection (natural or synthetic), culture conditions (differentiated or undifferentiated cells, growth factors, and extracellular matrix (ECM)), and 3D construct development (Cui et al., 2017). Bone-cartilage interface regeneration involves the cartilage and subchondral bone. Indeed, this technology has been clinically applied to assist in OCAs (Okoroha et al., 2018; Huotilainen et al., 2019; Russo et al., 2021) via 3D scanning and printing of the defect site to determine the size of patient-specific allograft plugs prior to grafting. However, a single 3D printed scaffold for bone or cartilage does not achieve the goal of osteochondral interface regeneration. A full understanding of the structure and composition of bone and cartilage, as well as of the reconstruction process is necessary to achieve regeneration of the osteochondral interface via 3D printing. The osteochondral interface is a specialized area that connects two tissues with different biochemical and mechanical properties. The transition of mechanical loads between cartilage and bone owe to osteochondral interface structure (Yang and Temenoff, 2009). The osteochondral interface is typically <1 mm and contains three orders of magnitude (quantum) of mechanical strength differences in addition to gradient variations in growth factor concentrations and cell differentiation. For 3D printing, the scaffold must achieve a spatially graded mechanical and chemical mimicry in the sub-millimeter range, which in turn complicates the design of the scaffold and its subsequent selection of cells and growth factors, as each region has different optimal conditions. Therefore, to successfully regenerate the osteochondral interface, the interdependent nature of the interfacial structures must be considered to attain the best balance of mechanical and biological properties.

Based on the speedy development of TE and regenerative medicine, various 3D printing regeneration plans have been schemed for osteochondral interface. The scaffold is the fundamental basis for 3D printed regenerative osteochondral interfaces, hence, good biodegradability and histocompatibility must be achieved in scaffold materials (Fu et al., 2018). Currently, natural materials (Bonani et al., 2018), synthetic materials (Frassica and Grunlan, 2020), ceramics (Wen et al., 2017), glass (Brauer, 2015), and composite materials (Turnbull et al., 2018), are used to construct scaffolds. Additionally, the method of material binding significantly impacts the structural strength of, and cell attachment to, the scaffold (Aisenbrey et al., 2018; Gao et al., 2020). Based on the multilayered hybrid structure of osteochondral bone, 3D printing research has focused on efforts to form layered structures that mimic the natural osteochondral interface. For example, Li et al. (2018b) designed a hybrid scaffold of hydroxyapatite (HAp), polylactic-co-glycolic acid (PLGA), and extracted bovine cartilage matrix that adequately mimicked the natural tissue structure. The healing of damaged tissues requires effective cell implantation and survival, thus, the ability of cells to be delivered on 3D-printed scaffolds and, subsequently, adhere and survive at the targeted site, improves the success of tissue regeneration (Cui et al., 2020). Specific cells have the potentiality to differentiate into target cells. For example, mesenchymal stem cells (MSCs) can differentiate into chondrocytes *in vitro*, while transforming growth factors (TGF), and growth factor (GF) signaling, are responsible for regulating the differentiation of mesenchymal cells into chondrocytes and the eventual formation of cartilage tissue (Foster et al., 2015). Additionally, the culture substrate must effectively promote cell proliferation, delay chondrocyte dedifferentiation without further ossification (i.e., endochondral ossification), and suppress the expression of genes involved in chondrocyte hypertrophy (Yang et al., 2018). To achieve this, 3D printing of regenerative osteochondral interfaces often includes growth factors, transforming growth factors, among other materials (ECM, metal ions, etc.) (Deng et al., 2018; Saha et al., 2013). Considering that the bone-cartilage interface structure is surrounded by cartilage and subchondral bone, all of which have their own structural layers, current research is concentrated on the development of multi-factor combinations and advanced delivery methods for reliable osteochondral tissue regeneration (Han et al., 2015).

**FIGURE 1:**
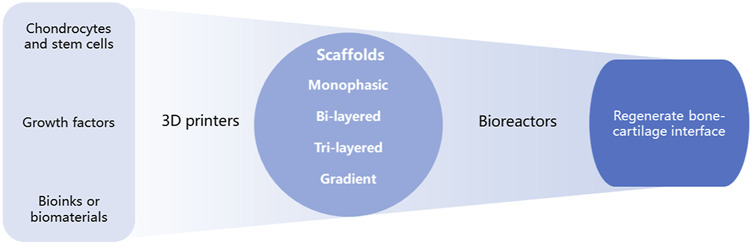
Schematic process of 3D printing for regenerating bone-cartilage interface.

Bone-cartilage interface regeneration has been neglected due to its complexity. With the importance of the bone-cartilage revealed, many researchers have started to focus on it. Thus, compared with the previous reviews, we want to have a comprehensive summary about the 3D printing for bone-cartilage interface regeneration and update the advanced progress. This review highlights new developments regarding materials, cells, signaling molecules, and the latest scaffold designs for 3D printing at the osteochondral interface by providing an overview of osteochondral structures, OCD, and repair mechanisms of osteochondral structures. We then present the current challenges and future directions in this field to support the development of effective 3D printing methods for osteochondral interface regeneration (Figure 1).

## 2 Osteochondral

### 2.1 Organizational Structure

**FIGURE 2:**
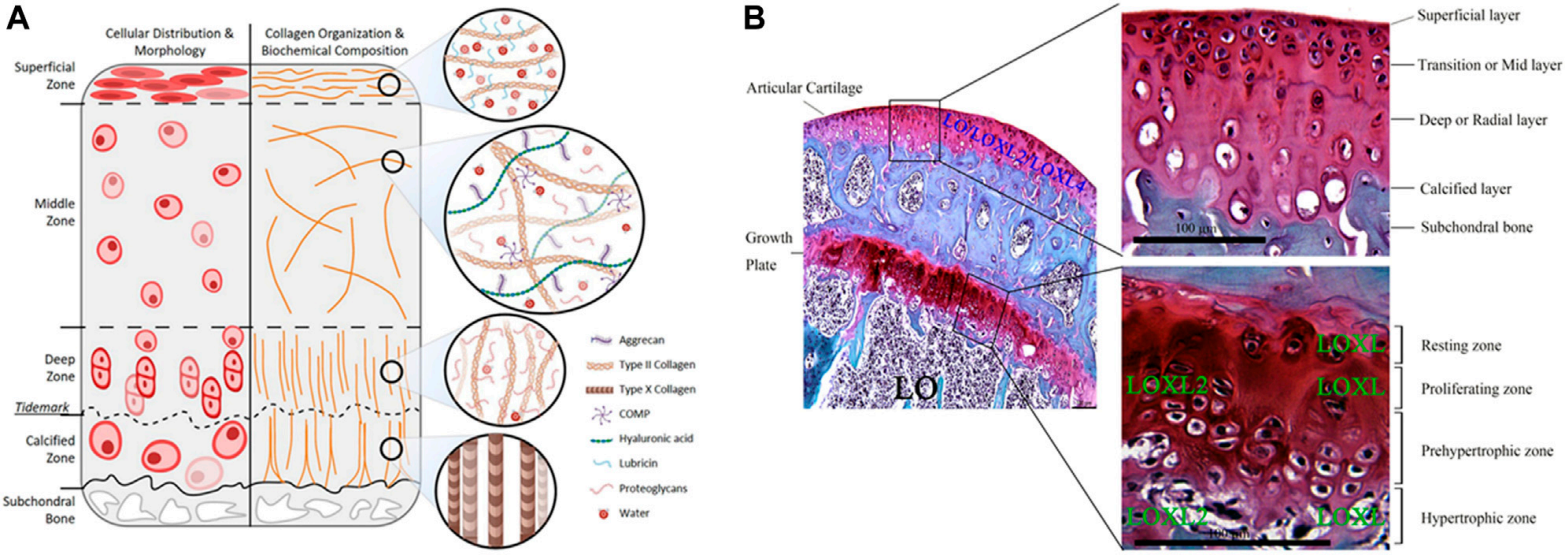
Schematic representation and histological images of osteochondral structures. **(A)** Schematic representation of articular cartilage and the gradient distribution structure. Reproduced with permission (Thorp et al., 2021). Copyright: ^©^2021 by the authors. **(B)** Safranin O-Fast Green staining shows the different expression of LO, LOXL and LOXL2 (scale bar = 100 µm). Lysyl oxidase: LO; lysyl oxidase like-2: LOXL2; Lysyl oxidase-like enzymes: LOXL. Reproduced with permission (Lin et al., 2020). Copyright: ^©^2020 Lin, Xu, and Li.

Osteochondral tissue comprises the cartilage layer, bone-cartilage interface, and subchondral bone (Figure 2). The cartilage in the uppermost layer is essential for joint function since it is responsible for lubrication, protection, and weight-bearing. Damage to cartilage caused by frictional forces can lead to degenerative lesions that destroy the osteochondral interface and extend to the subchondral bone. Cartilage is distributed throughout the body, such as in the external ear, nose, tracheal walls, ends of helper bones, and between the bones of the spinal roots. There are three types of cartilage: hyaline cartilage, fibrocartilage, and elastic cartilage, of which the hyaline cartilage is the most widely distributed in the body, and contains the articular cartilage. The articular cartilage is an interconnective tissue that covers the epiphyseal surface of the joint. It is a biphasic medium containing approximately 80% water, contains no nerves or blood and lymphatic vessels, uncapable of self-regeneration, and has only a single cell type, the chondrocytes (Jiang and Tuan, 2015; Armiento et al., 2018). Chondrocytes primarily produce ECM and balance its content, thereby maintaining the microenvironment around the cartilage. Although cartilage composition may seem simple, its complex biomolecular roles, multilayered hierarchical structure, and specific tissue functions hinder its regeneration. The structure and content of the cartilage’s ECM is a major determinant of normal function, while its components play different, but related roles (Krishnan and Grodzinsky, 2018). The proteoglycans of ECM make up about 5%–10% wet weight of cartilage tissue, while proteoglycans are dominated by aggrecan, which contains high levels of glycosaminoglycans (GAGs), including HA and chondroitin sulfate. GAGs are negatively charged and attract cations, creating ion-induced osmotic swelling, while the large amount of water absorbed provides the ECM with compressive stiffness, which significantly contributes to the weight-bearing mechanism of articular cartilage (Katta et al., 2008; Moshtagh et al., 2018). Collagen fiber is another important source of organizational strength, which is primarily composed of collagen type II. Nevertheless, the IV, VI, IX, X, XI, XII, XIII, and XIV account for only a small part of the mature matrix, but they have specific biological functions as well as act a pivotal part in the mechanical properties, organization, and shape of articular cartilage (Luo et al., 2017). The collagen fibril network contains numerous GAG chains and proteoglycan-bound aggregates of 300 MDa, interlaced by structured collagen (Athanasiou et al., 2009; Bajpayee and Grodzinsky, 2017).

Articular cartilage is highly organized and consists of four zones: superficial (tangential), intermediate (transitional), deep (radial), and calcified (Carballo et al., 2017). Numerous cells of the articular cartilage, ECM, and collagen fibers are anisotropic, especially in the superficial zone, where collagen fibers have a preferential orientation (Hossain et al., 2020), which is the main obstacle in regenerating the osteochondral interface.

The superficial zone, located at the surface, has the following distinct structural features: 1) Collagen fibers are aligned parallel to the joint surface and have lower proteoglycan content and fixed charge density compared with deeper tissues. 2) Contains an extensive network of elastic fibers roughly aligned with the collagen fibers in a plane parallel to the surface. 3) The superficial chondrocytes are disk-like shaped. 4) The ECM contains elastin and lipids. 5) SFZ cells produce a protein responsible for joint lubrication, which is encoded by *PRG4* and helps to protect the articular cartilage (Rolauffs et al., 2010; Mansfield et al., 2015; Xuan et al., 2019). The largest region, the middle zone, has rounded central chondrocytes; the type II collagen (Col II) is randomly distributed in the ECM (Amanatullah et al., 2014). The middle zone withstand compression and recover from the impact on the articular surface owing to these properties. The deep zone is distinct from the surface zone, with spherical chondrocytes, collagen fibers aligned obliquely to the articular surface, and a lower cell content but higher compression modulus. The border between the calcified and deep zones forms a distinctive line on the transverse light microscopic sections, called the tidemark, which marks the transition from the deep zone to the calcified zone (Mansfield and Winlove, 2012). The calcified zone contains a small number of mast cells (Diederichs et al., 2018) capable of secreting type X collagen (Kirsch and von der Mark, 1991), the mineralizing enzyme alkaline phosphatase (ALP), the HAp binding protein osteopontin, and MMP13 (Gannon et al., 1991; Hoemann et al., 2012). Collagen fibers in the calcified areas are arranged in an arch shape and contribute to the reinforcement of cartilage tissue. The strong interadhesion and intermediate stiffness of calcified cartilage facilitate load transfer, prevent cartilage delamination, and serve as a transition between plastic cartilage and stiff subchondral bone.

The subchondral bone, located beneath the cartilage, is formed by the subchondral plate and a 6 mm layer of trabeculae (also known as the subarticular spongiosa) (Henrotin et al., 2012). The subchondral bone plate is immediately below the calcified cartilage layer and a thin cortical layer (Milz and Putz, 1994). The articular cartilage separates from the bone marrow on account of a unit formed by the two mineralized layers of the subchondral plate form (Madry et al., 2010). The subchondral plate is a permeable structure with distinct pores that provide a direct link between the articular cartilage and subchondral tuberosity. Arterial and venous vessels and nerves penetrate the channels and send tiny branches into the calcified cartilage. Vessels distribution depends not only on the amount of intra-articular stress, but also on the stress variations in different joints (Holmdahl and Ingelmark, 1950; Madry et al., 2010). The subchondral cancellous bone is more porous, while the volume, density, and stiffness are lower than those of the cortical plate (Sharma et al., 2013). The permeability of both provides nutrition and timely physiological and pathological feedback to the cartilage. The thickness of the subchondral bone plate varies depending on the joint, while there is a regional specificity in the thickness and density distribution of the subchondral bone plate. The resulting bone trabeculae are referred to as “supporting trabeculae” (Madry et al., 2010). Cartilage is the load-bearing and protective structure of joints. However, cartilage cannot bear weight alone due to its limited regenerative capacity, whereas subchondral bone is considered a weight-bearing structure with good regenerative capacity. Thus, the osteochondral unit should be used to withstand physiological loads, allowing physiological and structural balance (Hoechel et al., 2012; Goyal et al., 2017). Meanwhile, the sodium citrate (SC) bone acts as a dynamic component of the OC unit, transmitting forces through the joint and adapting to its mechanical demands (Hoechel et al., 2012). The specific structure of the subchondral bone helps to minimize and redistribute axial forces, cushion shock through deformation and during stress transmission to avoid excessive stress damage to the cartilage. After loading, the subchondral bone can be regulated, by the blood vessels and nerves between the pores, to induce the release of joint fluid, proteoglycans, and fibers (Goyal et al., 2017). Subchondral bone is also a repository of stem cells, with subchondral bone providing undifferentiated bone marrow stem cells as the sole source for new chondrocyte generation. Various growth factors are also provided by subchondral bone, and play an active role in cartilage healing and remodeling (Goyal et al., 2017).

**FIGURE 3:**
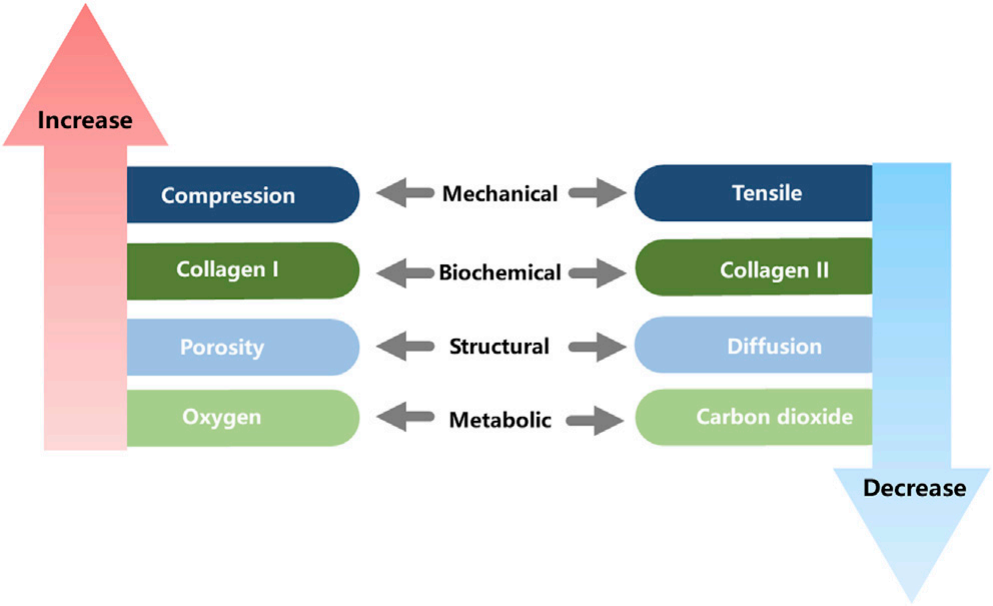
Gradient change of osteochondral properties.

Many of the functional properties of the joint arise from the unique gradient structure of the osteochondral unit (Figure 3). From the tip (cartilage) to the base (bone), the following changes occur: biomechanical compression and elastic modulus gradually increase, while hydrostatic pressure and viscous modulus gradually decrease. Additionally, hydroxyapatite (HAp) and collagen type I (Col I) contents gradually increase, while water and collagen type II (Col II) gradually decrease. Structurally, vascularity, permeability and porosity gradually increase. In terms of bioelectricity, piezoelectricity and pyroelectricity gradually increase, while flow potential, dielectric constant and diffusion potential gradually decrease. Finally, metabolically, glucose and oxygen contents gradually increase, while carbon dioxide and lactic acid gradually decrease (Zhou et al., 2020).

### 2.2 Osteochondral Defects

Avascular articular cartilage cannot form blood clots nor does it engage in necrosis, inflammation, repair, or remodeling, all of which are required for healing injured tissues. As such, articular cartilage has a limited intrinsic healing and repair capacity, and mature chondrocytes are not sufficient to produce adequate ECM (Carballo et al., 2017). With age, chondrocyte apoptosis, water content, and proteoglycan size decrease within the ECM, leaving articular cartilage highly vulnerable to damage (Sophia Fox et al., 2009). In addition, anti-inflammatory treatments, diabetes, and menopause have been shown to disrupt cartilage structure and affect cartilage stiffness, leading to greater susceptibility to cartilage destruction. As the lack of continuous collagen fibers of the transition from calcified cartilage zone to subchondral bone plate, the bone-cartilage interface is more fragile than the transition within the cartilage zone in the structural property. Thus, the bone-cartilage interface is susceptible to damage when the cartilage is damaged. In healthy bones, a balance exists between bone resorption and deposition in response to dynamic adaptation to mechanical loading. In Osteochondral Defects (OCD), this balance is disrupted, leading to changes in the structure of the subchondral trabeculae (T bone), including increased thickness of the subchondral bone plate, formation of new bony structures at the joint edges (osteophytes), and development of subchondral bone cysts (Goldring and Goldring, 2010; Loeser et al., 2012; Funck-Brentano and Cohen-Solal, 2015; Tuerlings et al., 2021).

Cartilage defects can be classified, using various methods, according to severity, width, and depth, according to different methods. The most popular approach is to group patients based on the Outerbridge classification system, which classifies cartilage areas on a 0–IV scale. Grade 0: healthy cartilage; Grade I: softened and swollen cartilage, usually requiring probes or other instruments for diagnosis; Grade II: partial-thickness defect with a defect ≤1.5 cm in diameter or reaching the subchondral bone; Grade III: cartilage defect >1.5 cm in diameter or reaching the subchondral bone; Grade IV: exposure of the subchondral bone (Outerbridge, 1961, Outerbridge, 1964; Slattery and Kweon, 2018). Alternative grading systems that can accurately assess the degree of cartilage damage, include the International Cartilage Repair Society (ICRS), Oswestry Arthroscopy Score (OAS) (van den Borne et al., 2007), Histology/Histochemistry Grading System (HHGS), and Osteoarthritis Research Society International (OARSI) Cartilage Histopathology Assessment System (OOCHAS) (Custers et al., 2007).

### 2.3 Osteochondral Repair Mechanisms

Cartilage heals through chondrocyte secretion of ECM and fibrosis. Subchondral bone remodeling is an important regulatory mechanism by which bone tissue adapts to changes in the local biological microenvironment and mechanical stimuli, and includes, in sequence, the resorption phase (initiation/activation of bone remodeling at a specific site), reversal phase (bone resorption and simultaneous recruitment of MSCs and osteoprogenitor cells), and osteogenic phase [osteoblast differentiation and function (osteoid synthesis), and quiescent phase (completion of bone-like mineralization and bone reconstruction)]. During remodeling, bone resorption and bone formation are coupled, and the synergistic activity of osteoclasts and osteoblasts promotes the resorption of old bone tissue and subsequent new bone formation (Feng and McDonald, 2011). The mechanism of OCD healing has been studied in several animal models, and Shapiro et al. (1993) described the sequence of healing a 3 mm diameter OCD in the femoral rotor of a rabbit, including fibrin production, mesenchymal cell aggregation, cartilage formation, and bone formation. The mechanism of osteochondral repair in sheep is endochondral ossification, however, unlike rabbits, no evidence of MSC recruitment was found for OCD healing in the sheep model (Lydon et al., 2019).

## 3 3D Printing

### 3.1 Bioinks

Bioink is the base material for scaffold formation and are generally flowable liquids that can be easily squeezed and rapidly solidified, retaining their shape by physical or chemical stimulation (Dai et al., 2020). The porous structure, adjustable mechanical properties, and high water content can provide an appropriate environment for different cells to mimic the ECM. Furthermore, these inks can be easily loaded with bioactive molecules and cells to assist the adhesion, proliferation, and differentiation of target cells (Ozbolat and Hospodiuk, 2016).

#### 3.1.1 Natural Bioinks

Natural bioinks have a high water content, good biocompatibility and biodegradability, and the ability to transport metabolic waste and nutrients, which are critical for *in vivo* applications. When liquid-like natural inks undergo gelation, the loaded cells can be encapsulated in a three-dimensional structure. Natural bioinks used for osteochondral interface regeneration include collagen (Marques et al., 2019), gelatin (Echave et al., 2019), silk fibroin (Ni et al., 2020), silk sericin (Naskar et al., 2021), fibrin (Nulty et al., 2021), keratin (Shavandi et al., 2017), chitosan (Shoueir et al., 2021), alginate (Chen et al., 2018), HA (Yontar et al., 2019), and gellan gum (Choi et al., 2020) among other polysaccharides. However, the drawbacks of natural bioinks are their weak mechanical properties and antigenicity. Crosslinking can make up for these shortcomings (Lin et al., 2021), the common crosslinking strategies including light (Lee et al., 2020), UV (Frieß et al., 2021), energy electron irradiation (Tang et al., 2021) and enzymatically crosslinking (Wu et al., 2022) methods. Therefore, most natural bioinks used for 3D printing have improved mechanical properties by crosslinking (physically or chemically) and compounding synthetic polymers (Chawla et al., 2020). Alternatively, gelatin methacrylate (GelMA) is a photosensitive biohydrogel material obtained from methacrylic anhydride and gelatin that is often used for bone-cartilage repair (Gao et al., 2021) as a common alternative to natural bioinks.

The decellularized osteochondral ECM is also the focus of current research. Lin et al. (2018) prepared a decellularized ECM scaffold with natural components (mainly collagen) and three-dimensional tissue structures with good biocompatibility *in vitro* and *in vivo*. In this study, the biphasic scaffold was nearly devoid of angiogenesis, avoiding endochondral ossification due to vascular invasion into the cartilage region, and also had the ability to promote MSC proliferation and differentiation, as well as low immunogenicity, thus, successfully promoting regeneration of osteochondral tissue.

#### 3.1.2 Synthetic Bioinks

The wide variety of synthetic bioinks allows for diverse chemical and mechanical applications. Sequence modification can modulate the degradation rate of biodegradable polymers and influence the material properties of bioink solubilization and gelation (Austin and Rosales, 2019). Popular biodegradable synthetic bioinks include poly (caprolactone) (PCL), PLGA, and poly (lactic acid) (PLA) (Critchley et al., 2020). Although synthetic bioinks have stronger mechanical properties and printability than natural bioinks, they also have poor biocompatibility and biodegradability. Therefore, future research strategies should focus on establishing an effective combination of natural and synthetic bioinks to exploit the advantages of both materials, while providing possibilities for osteochondral regeneration solutions. Indeed, Guo et al. (2021) recently published a study on PCL-peptide complexes, in which they employed aqueous click conjugation to combine acetylene-capped PCL and peptides with different chemical characteristics and different chemical and biological origins. They then performed multi-material segmental printing using melt extrusion printing to generate a PCL-peptide scaffold obtained by μCT that maintained good printability. Moreover, *in vitro,* the scaffolds incorporating different tissue-specific peptides showed strong bioactivity and effectively promoted osteogenic or chondrogenic ECM deposition of bone marrow-derived MSCs (BM-MSCs) (Guo et al., 2021).

#### 3.1.3 Bioceramics, Bioglass and Biological Composites

Bioceramics were originally developed for the repair, reconstruction, and replacement of diseased hard tissues (e.g., teeth and bone) and were later adapted for artificial heart valves, artificial tendons, etc. Bioceramics promote biomineralization and have the advantages of good wear resistance, osteoconductivity, corrosion resistance, hard surface, oxidation resistance, and low coefficient of friction (Hasan et al., 2013). Bioceramics can be further divided into natural and synthetic bioceramics, that is, bioinert ceramics (e.g., Al_2_O_3_, ZrO_2_, etc.), bioactive glasses (e.g., dense hydroxyapatite), glass ceramics, and bioresorbable calcium phosphate substrates (Pina et al., 2018). Previous studies have showed that ceramics, such as HAp), or other calcium phosphate (Ca-P) ceramics (including tricalcium phosphate, (TCP)) or bioactive glasses, play an important role in the promotion of the formation of bone-like apatite layers on the surface of scaffolds upon implantation. This is considered a positive feature of bioceramic bone binding, which improves the stability of implant fixation (Mano and Reis, 2007). In addition, the surface of bioceramic scaffolds can absorb osteoinductive factors and/or ions and continuously release them to modulate the surrounding environment, promoting the differentiation of MSCs and thus bone formation *in vivo* (Ma et al., 2018).

β-TCP is one of the most widely used and effective bioceramics and has good osteoconductive and osteoinductive properties. Kosik-Koziol et al. (2019) investigated the effect of different concentrations of TCP on the efficiency of UV-induced crosslinking of GelMA and concluded that 0.5% w/v β-TCP was optimal for forming ideally shaped scaffolds with calcified cartilage development-related biological properties at the optimal concentration (Kosik-Koziol et al., 2019).

**FIGURE 4:**
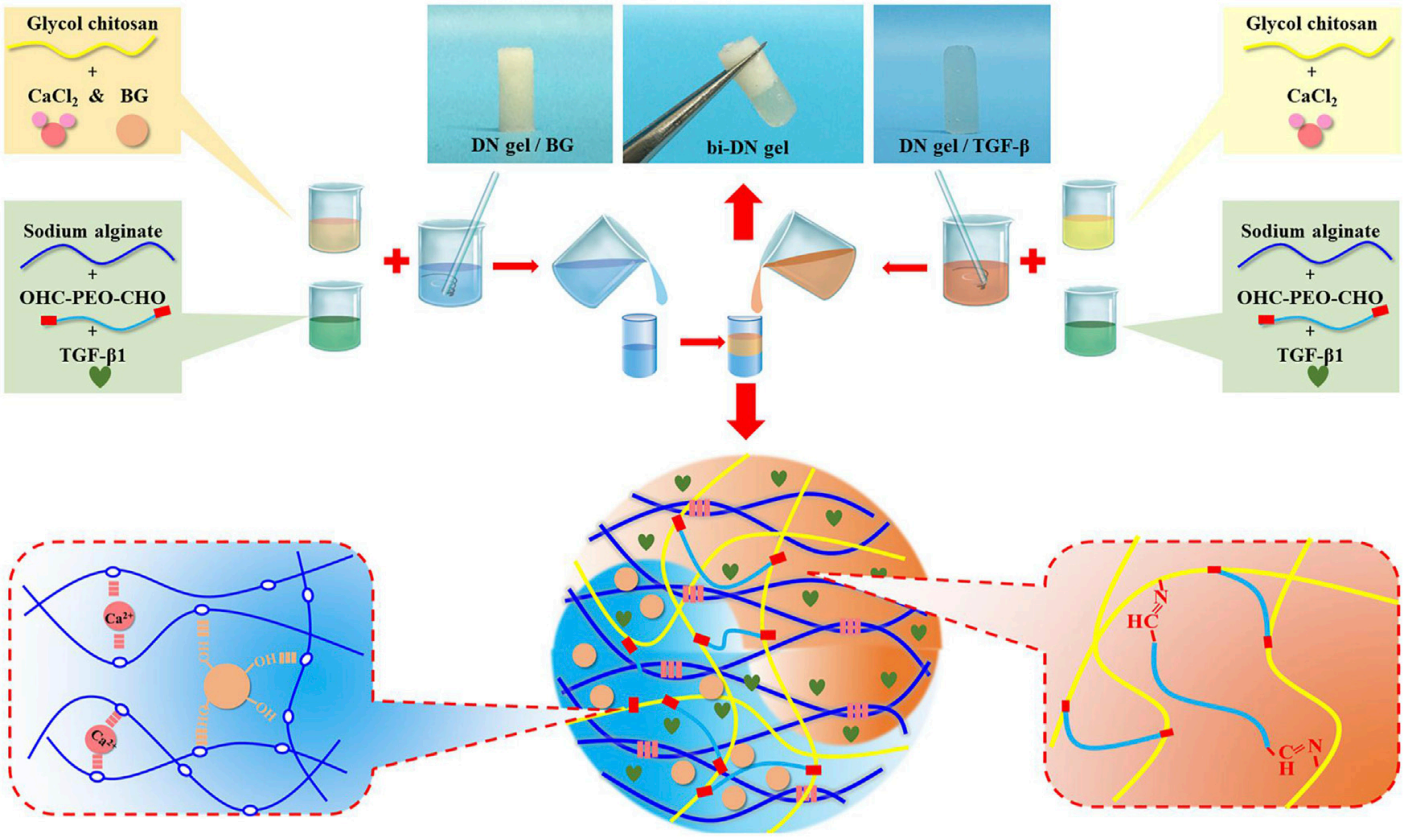
Schematic illustration of the composite bioinks (bi-DN gel). DN, double-network; CaCl_2_, calcium chloride; BG, bioactive glass; TGF, transforming growth factors; Reproduced with permission (Liu et al., 2020). Copyright: ^©^2020 Liu, Zhao, Zhu, Gao, Ye, Zhou, Qiu, Wang, Tian and, Qu.

The addition of silicon (Si) (Yu et al., 2018), strontium (Sr) (Deng et al., 2018), Molybdenum (Mo) (Dang et al., 2018), lithium (Li) (Chen et al., 2019), Copper (Cu) (Lin et al., 2019), or other elements, can also improve the biological properties of scaffolds. Biological composites are the most suitable option for treatment of osteochondral interface injuries (Figure 4). Biocomposites exhibit excellent mechanical properties and bionic properties owing to their highly organized, heterogeneous structure across various length scales (Rajasekharan et al., 2017). Indeed, You et al. (2018) obtained ALG/HAP composites by homogeneous dispersion of HA in sodium alginate (ALG) hydrogel using SC. The ALG/HAP composite scaffold promoted chondrocyte secretion of type X collagen and increased ALP activity and mineral deposition (You et al., 2018). We believe that composites that combine the advantages of different materials will be the key for developing effective 3D printing strategies for regenerative osteochondral interfaces.

### 3.2 Chondrocytes and Mesenchymal Stem Cells

When creating a scaffold for osteochondral interface regeneration, the addition of specific cells to the scaffold can alter the way the surrounding tissues interact with the scaffold, which in sequence can affect the way the overall healing occurs. Scaffolds often carry several cellular components, the most common of which used for osteochondral interface regeneration are chondrocytes and stem cells. Chondrocytes are tissue-specific cells and, in diseased articular cartilage, do not proliferate sufficiently. Thus, chondrocytes adhering to the scaffold can help regenerate functional cartilage tissue at the defect site. Meanwhile, stem cells can self-renew and differentiate into multiple mature cell types; among these, MSCs are the most promising for osteochondral repair as they can differentiate into bone or cartilage under specific induction conditions (Vasiliadis and Galanis, 2020).

Chondrocytes are often used to study the effect of scaffolds on chondrocyte proliferation and maturation *in vitro* (Deng et al., 2018), and to place chondrocytes in scaffolds to help regenerate cartilage tissue. Experiments were mostly performed with bovine (Wuest et al., 2018), rabbit (Zhou et al., 2017), rat (Bao et al., 2020) and human (Takahashi et al., 2018) origin chondrocytes. Primary monolayer chondrocyte cultures can generate numerous Col II and cartilage-specific proteoglycans (Perka et al., 2000). Primary culture chondrocytes are of limited origin, however, multiple *in vitro* passages cause them to lose their phenotype and convert to fibroblasts, reducing Col II expression and increasing type I collagen expression at the mRNA and protein levels. Sliogeryte et al. (2016) monitored innovativly isolated primary chondrocytes (P0) and cells of the first generation (P1) cultured in monolayer culture for 9 days. They found that monolayer culture and dedifferentiation strengthen membrane-actin cortex adhesion and increase cortical F-actin organization and ERM protein expression (Sliogeryte et al., 2016). These changes influence chondrocyte functions, including migration, endocytosis, and differentiation (Sliogeryte et al., 2016).

As such, research has focused on isolating primary chondrocytes from cartilage. However, chondrocytes are usually digested with collagenase to facilitate complete isolation, which can be detrimental to the cells. That is, too much or too little collagenase can lead to failure or low yield (Lepage et al., 2019). Hence, in a study conducted by Muhammad et al. (2019), a protocol for chondrocyte isolation was optimized using trypsin-ethylenediaminetetraacetic acid (EDTA), collagenase II in Hank’s balanced salt solution (HBSS), and collagenase II in Dulbecco’s modified Eagle medium/Nutrient Mixture F-12 (DMEM/F-12) for chondrocyte isolatio. They found that collagenase II in HBSS retained the chondrogenic phenotype, especially proteoglycan expression (Muhammad et al., 2019). Meanwhile, for chondrocyte dedifferentiation, Jeyakumar et al. (2017) demonstrated the positive effect of platelet-rich plasma (PRP) on proliferation and redifferentiation of dedifferentiated chondrocytes, and concluded that the standard usage of 10% FCS could be replaced with 10% PRP.

In addition, many researchers have used MSCs as seed cells. BM-MSCs were the first MSCs used for bone and cartilage repair, however, collection of autologous BM-MSCs was highly invasive for the patient. Alternatively, adipose-derived mesenchymal stem cells (AMSCs) are relatively noninvasive, easy to obtain, and have demonstrated differentiation potential in specific settings (Yamasaki et al., 2019). Human turbinate-derived mesenchymal stromal cells (hTMSCs) are MSCs with chondrogenic, osteogenic, and lipogenic differentiation potential (Hwang et al., 2012). hTMSCs were used by Shim et al. as seed cells encapsulated in cucurbit [6] uril (CB)/1,6-diaminohexane (DAH)-supramolecular HA in a multilayer 3D scaffold. hTMSCs with ALP, collagen I (Col I), and osterix (Osx) were not significantly expressed, whereas the expression of aggrecan (ACAN), collagen type II (Col II), and SRY-related high-mobility-group box 9 (Sox-9) was enhanced (Shim et al., 2016).

Umbilical cord blood mesenchymal stem cells (UCB-MSCs) have the general characteristics of MSCs. However, unlike hTMSCs and BM-MSCs, UCB-MSCs have the highest amplification potential and possess osteogenic and chondrogenic differentiation capacity, without adipogenic differentiation capacity. Moreover, UCB-MSCs exhibit lower expression of immunogenic markers (CD105 and CD90), are easily accessible from cord blood, and their use is not ethcially controversial. Indeed, Zheng et al. (2019) demonstrated that UCB-MSC xenografts contributed to osteochondral repair in a rabbit model.

### 3.3 Growth Factors

Chondrocytes carried by the stent or migrating from the tissue surrounding the damaged site usually produce fibrocartilage tissue in the absence of growth factors, rather than the ideal hyaline cartilage tissue. Growth factors are naturally occurring substances, such as hormones or proteins that can cue and expedite cell growth in a certain direction and are useful. In osteochondral regeneration, specific growth factors can induce the differentiation of stem cells into chondrocytes. For example, factors such as bone morphogenetic proteins (BMPs), insulin-like growth factors (IGFs), and transforming growth factors (TGFs) have been shown to promote the differentiation of stem cells into cartilage or osteogenesis (Mora-Boza and Lopez-Donaire, 2018). Vascular endothelial growth factor (VEGF) and BMP-4 promote angiogenesis for nutrient transport, oxygen exchange, waste transport, etc. (Lee et al., 2020). The synthesis and modification of collagen in chondrocytes are controlled by the metabolism of HIF-1α (Stegen et al., 2019). Growth factors can also influence the physical properties of nascent cartilage tissues. And there is an interesting phenomenon that the expression of BMP requires the expression of SOX genes, which in turn promotes the expression of SOX genes during chondrogenesis. Within a rabbit model of OCD in the patellar groove, the addition of TGF-β1 and IGF-1 induces BM-MSCs to differentiate into chondrocytes and increase matrix synthesis, enabling the formation of smooth-surfaced hyaline cartilage at the defect site (Gugjoo et al., 2020).

PRP is a concentrate prepared from fresh blood by low-speed centrifugation and contains large amounts of autologous growth factors, including platelet-derived growth factor (PDGF), TGF-β, IGF, epidermal growth factor (EGF), and VEGF. PRP stimulates chondrocyte proliferation and promotes the production of therapeutic cells in cartilage tissues. In addition, PRP induces autocrine growth factors to promote cartilage healing (Chang et al., 2018). Jiang et al. (2021) suggested that PRP can promote osteochondral regeneration by promoting the polarization of M2 macrophages. Accordingly, they prepared PRP-GelMA hydrogel scaffolds inoculated with rabbit BMSCs and observed an increase in M2 macrophage, which had an anti-inflammatory effect and provided a favorable environment for osteochondral regeneration (Jiang et al., 2021).

Recently, Vainieri et al. (2020) investigated the effects of 50 and 100 ng/ml PDGF-BB, chemokine ligand 5 (CCL5/RANTES), and stromal cell-derived factor-1 (SDF-1) on the migration of bone marrow mesenchymal stem cells (BMSCs) *in vitro*; the migration distance of BMSCs in three-dimensional spheroids was examined by confocal microscopy. All groups, save for 100 ng/ml RANTES, promoted BMSC migration *in vitro*, with 50 ng/ml PDGF-BB being the most effective (Vainieri et al., 2020).

BMP-2 and VEGF can promote osteogenesis and angiogenesis at the osteochondral interface. However, they are natural macromolecules that are unstable and expensive. As an alternative, Wang et al. (2021) proposed that synthetic osteogenic peptide (OP) and angiogenic peptide (AP) could be used. Indeed, scaffolds containing AP and OP exhibited rapid release of AP and sustained release of OP inducing significant vascularity and new bone formation, respectively (Wang et al., 2021). Furthermore, since the osteochondral interface involves both cartilage and subchondral bone, researchers have focused on the use of biphasic scaffolds carrying osteogenic and chondrogenic peptides, respectively. For instance, osteogenic peptide/TGF-β1 (Wang et al., 2020) and HA bind (hyaluronic acid-binding peptide)/E3 (mineralizing peptide) (Camacho et al., 2021) have been successfully used for osteochondral tissue regeneration.

Collectively, these studies suggest that growth factors act a crucial part in influencing the effectiveness of stem cells in regenerating tissues. In addition to typical growth factors, biomolecules with different functions have great potential. For example, Zhu et al. (2020) compared polyethylene glycol diacrylate (PEGDA)/ECM scaffolds with PEGDA/ECM/honokiol (an inflammatory phytomolecule) scaffolds, and found the honokiol group showed significantly enhanced osteochondral regeneration 4 and 8 weeks postoperatively in rat model (Zhu et al., 2020).

Johnson et al. (2012) identified a small molecule, kartogenin, that promotes cartilage production by inducing the transformation of MSCs into chondrocytes. Kartogenin interrupts the interaction between filamin A (FLNA) and CBF-β and controls the expression of a family of proteins that play key roles in musculoskeletal development. Topical administration of kartogenin to mice with osteoarthritis-like symptoms, triggered the development of cartilage. (Johnson et al., 2012). Moreover, Zhao et al. (2020) prepared KGN-encapsulating PLGA microspheres using a solid-oil-water double solvent evaporation technique, complexed with CECM scaffolds containing TGF-β3, and demonstrated that the scaffold prolonged the activity of KGN and supported the adhesion, proliferation, and chondrogenic differentiation of BMSCs *in vitro*. Moreover, Zhao et al. (2020) reported the successful integration of new cartilage at the defect site with surrounding tissues in a rabbit femoral condylar cartilage defect model. This study provided novel insights regarding the generation of scaffolds with kartogenin, however, no positive synergy was observed between kartogenin and TGF-β3 (Zhao et al., 2020).

Finally, considering that ECM exosomes act a pivotal part in intercellular mitochondrial communication (Singh et al., 2017), Chen et al. (2019b) prepared a 3D printed cartilage ECM/GelMA/extracellular body scaffold with radial channels via desktop stereolithography. They found that ECM exosomes could restore chondrocyte mitochondrial dysfunction in a rabbit OCD model possibly associated with 10.3% of its internal mitochondria-associated proteins (Chen et al., 2019b).

### 3.4 Scaffold Design and Machining

**FIGURE 5:**
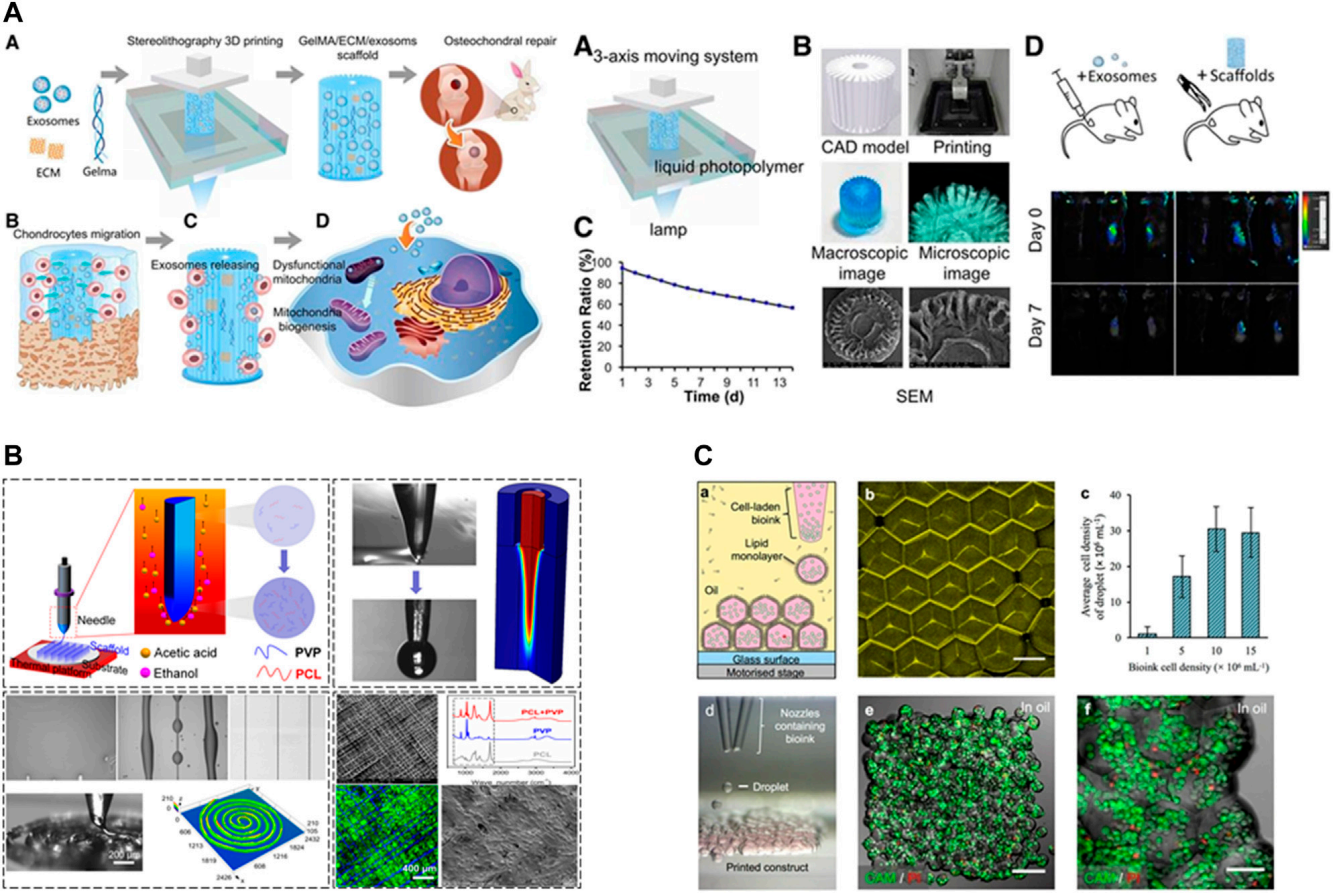
Recent new technologies for 3D printing. **(A)** desktop-stereolithography 3D printing. Reproduced with permission (Chen et al., 2019b). Copyright: ^©^2019 by Ivyspring International Publisher. **(B)** Tip-Viscid Electrohydrodynamic Jet (TVEJ) 3D Printing. Reproduced with permission (Li et al., 2021). Copyright: ^©^2021 by the authors. **(C)** 3D printing of aqueous droplets. Reproduced with permission (Graham et al., 2017). Copyright: ^©^2017 by the authors.

The role of the scaffold in the development of osteochondral tissue is to provide a shape for tissue regeneration and to load cells and bioactive factors. The advent of three-dimensional printing (3DP) technology has made it possible to fabricate highly complex scaffolds (Figure 5). An ideal scaffold must possess an appropriate pore size, interconnectivity, and surface topography, biocompatibility, vascularity, biodegradability, non-cytotoxicity, good mechanical and rheological properties, as well as a simple and economical preparation process.

#### 3.4.1 3D Printing Techniques

The most commonly used 3D printing technologies are fused deposition modeling (Distler et al., 2020), stereolithography (SLA) (Kumar and Kim, 2020), selective laser sintering (Zeng et al., 2020), inkjet (Li et al., 2020), 3D plotting (Seok et al., 2020) and LOM (Luong et al., 2018).

Mellor et al. (2017) pioneered the combination of electrospinning and 3D printing technologies to obtain a scaffold with the advantages of both. During implantation into a porcine osteochondral defect model, the nanofiber composite scaffold obtained by electrospinning alone was prone to delamination, whereas the composite micro/nanofiber scaffold did not peel off during culture, and the cells proliferated stably on days 1, 4, 7, and 21 (Mellor et al., 2017). Additionally, Graham et al. (2017) designed a novel droplet-based 3D printing technique that printed ≤200 μm high-resolution 3D geometrically shaped ovine MSCs. After 5 weeks of *in vitro* culture, the printed oMSCs differentiated into chondrogenic lineage cells, generating cartilage-like structures with Col II (Graham et al., 2017). Still further, Schoonraad et al. (2021) utilized digital light processing-based stereolithography (DLP) to print a bilayer scaffold. The prepared photoresins were printed in CAD files as 25 µm layers, irradiated at λ = 405 nm for 6 s followed by a brief rinse with 100% ethanol to remove redundant resin, and then heat cured in an oven at 120°C under vacuum for 1 h (Schoonraad et al., 2021). Li et al. (2021) investigated the application of tip-viscid electrohydrodynamic jet printing (TVEJ) for osteochondral regeneration. TVEJ utilizes a combination of thermal, flow, and electric fields to prepare PCL/PVP composite osteochondral scaffolds by viscous tip jets generated at the tip of the needle; the solvent evaporation rate was adjusted to allow flexible control of various printing patterns and structural resolution. The biocompatibility of the scaffold was demonstrated by *in vitro* culture of murine MC3T3-E1 Subclone14 cartilage cells with cell survival rates of 84%, 88%, and 91% after 1, 2, and 3 days, respectively (Li et al., 2021). Idaszek et al. (2019) designed a multi-material deposition system based on a microfluidic platform with a hybrid chamber and proved its feasibility for depositing continuous gradients of cells and materials in 3D structures with high shape fidelity, appropriate porosity, and cell viability (Idaszek et al., 2019).

When stem cells are used for tissue regeneration, the compression and shearing of cells by the scaffold can lead to cell damage or death (Manoukian et al., 2018). The loss modulus (G″), energy storage modulus (G) and loss angle tangent (G″/G′) are the main parameters that determine the results of extrusion uniformity, extrudability, and structural integrity printing (Chen et al., 2020). The loss angle tangent is inversely proportional to the extrusion pressure. As excessive squeeze pressure can damage cells loaded in bioinks, extrusion pressure should be controlled to maintain cell viability at the lowest possible loss rate (Abdollahiyan et al., 2020). Moreover, pore shape and porosity affect the permeability/diffusivity, degradation rate, and elastic modulus of the scaffold. Zhang et al. (2020a) investigated the effect of porosity and pore shape on the mechanical properties of the scaffold using a finite element method, and concluded that the Young’s modulus (overall mechanical properties of the scaffold) decreases with increasing porosity of the scaffold (Zhang et al., 2020a). Additionally, Reed et al. (2016) fabricated highly porous, hydrophilic chitosan-alginate (Ch-Al) scaffolds by 3DP and directional freezing, resulting microchannels parallel to the *Z*-axis and lamellar pores with 300 μm long and 50 μm in diameter. A porous pore zone with a diameter of 100 μm was visible in the bottom 500 μm of the scaffold, with a complete transition from the lamellar to the spherical pore zone (Reed et al., 2016).

In the development of osteochondral tissue scaffolds, new or combined 3D printing strategies are developed or improved to obtain reproducible bionic structures with controlled porosity, composed of different materials, spatially organized, and capable of delivering cells and growth factors in a controlled manner. Such scaffolds are designed to address specific aspects of osteochondral tissue, namely vascularization, deposition of calcium phosphate in predefined areas, directing regeneration in certain directions (by gradient delivery of factors or anisotropic porous structures), development of different tissues (i.e., OCD), or inhibition of calcification and cell adhesion.

#### 3.4.2 Monophasic Scaffolds

**FIGURE 6:**
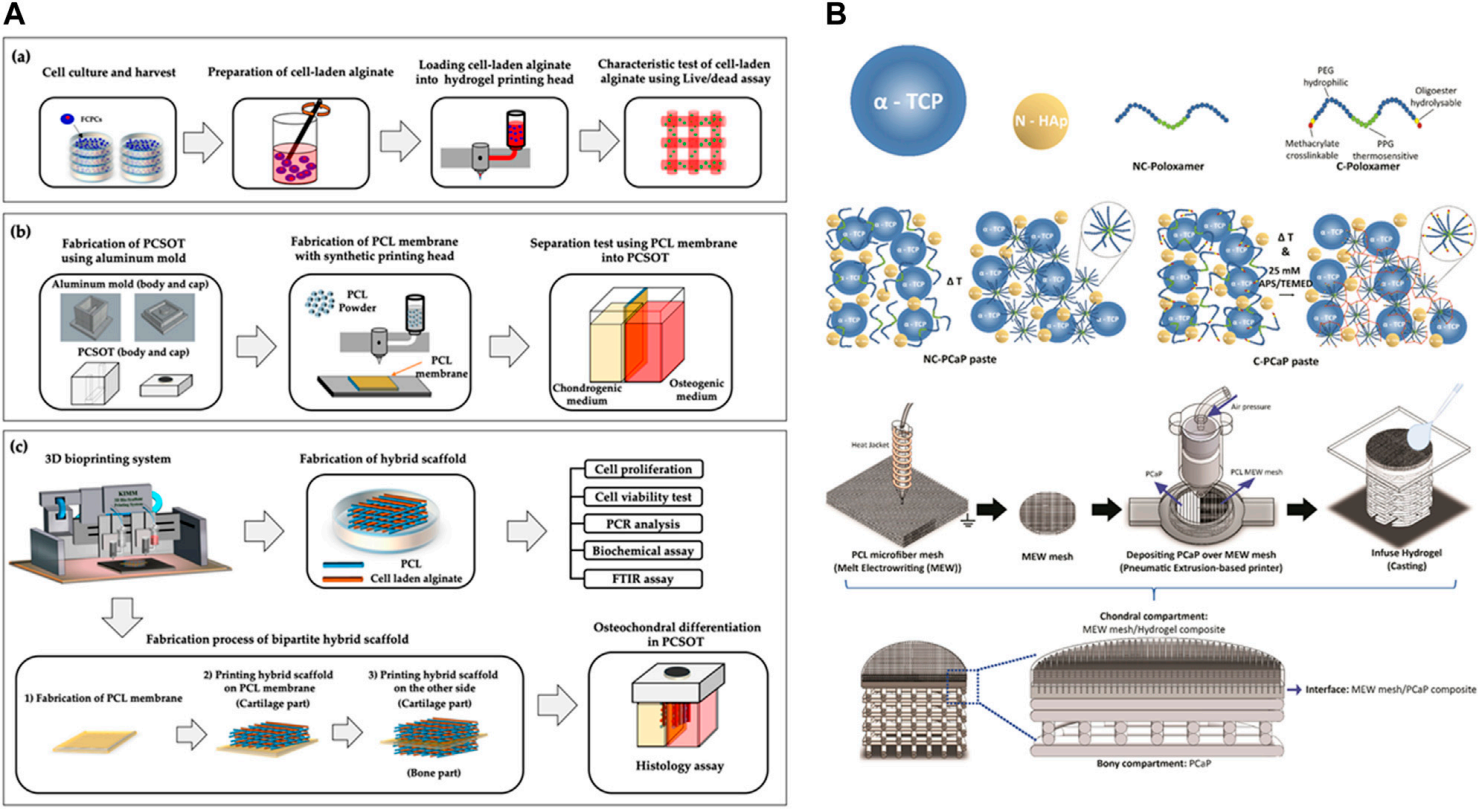
Representative design scheme of bi-layered and multi-layered scaffolds. **(A)** Polycaprolactone (PCL)/alginate bi-layered scaffold. Reproduced with permission (Yu et al., 2020). Copyright: ^©^2020 by the authors. **(B)** A multi-layered scaffold with bone layer (PCaP) connects with chondral layer (PCL) by the melt electrowriting mesh. Reproduced with permission (Diloksumpan et al., 2020). Copyright: ^©^2020 by the authors.

3D printing has long been used in osteochondral interface regeneration with monophasic scaffolds representing the earliest standard technique (Figure 6). Single-phase scaffolds use a single material with a single structure and porosity throughout; the same cell types and bioactive factors are distributed within the scaffold to accommodate the shape of the defect area. Studies of single-phase scaffolds have shown that they support the attachment and proliferation of chondrocytes and osteoblasts. However, due to the complex tissue composition and structure of the osteochondral interface, monophasic scaffolds do not simulate both cartilage and subchondral bone, let alone tidemark and cement lines.

#### 3.4.3 Bi-Layered, Tri-Layered Scaffolds

Current research focuses on hierarchical scaffolds, including bi-layered, tri-layered, and gradient layers (Zhou et al., 2019; Mancini et al., 2020).

Thunsiri et al. (2020) designed a bilayer biologically active biomaterial scaffold with a cartilage (AC) layer consisting of polylactic acid (PLA) and polycaprolactone (PCL) hybrid fibers printed in 3D and freeze-dried with chitosan (CS)/filamentous fibers (SF), as well as a bone layer consisting of PLA, PCL, and HA. Analysis of the mechanical properties showed that following culture of the AC layer scaffold with the human fetal osteoblast cell line hFOB1.19, and the B layer scaffold with SW1353 chondrocyte-like cells, increased cell survival was oberved in the AC and B layers, indicating that the presence of bioactive substances (CS and SF) promotes cell proliferation (Thunsiri et al., 2020).

Natarajan et al. (2021) printed a turbid solution containing PCL, PLGA, and chondroitin sulfate at a ratio of 65:30:5 at different filling densities to form a gradient cartilage layer. They used a dissolved-adhesion technology to bond the cartilage layer to the calcified layer to obtain a biphasic scaffold with simultaneous osteogenic differentiation potential. The resulting scaffold had a stable and continuous connection between the two layers at the interface. Moreover, approximately 35% of the bilayer scaffold (BLS) degraded after 3 months of immersion in PBS with synchronized precipitation and dissolution processes. Furthermore, by analyzing the viability of rabbit AMSCs, 3D printed scaffolds (i.e., PCL/PLGA/CS, PCL/PLGA/β-TCP, BLS) were found to have more live cells and fewer dead cells with no change in cell morphology after 3 days of culture. After 7 days of culture, the proliferation of AMSCs on composite scaffolds was significantly increased (*p* < 0.05) compared to that of the control. After 28 days of culture in the differentiation medium, AMSCs were active on the bilayer scaffold, and cells cultured on the scaffold containing CS and β had higher metabolic activity than those cultured in the control group. Most importantly, the CS- and β-TCP-containing BLS supported the differentiation of AMSCs into bone and cartilage cell lineages. At days 7 and 14, the ALP activity of BLS was significantly higher than that of the control (*p* < 0.05). On day 28, the GAG, collagen, and calcium contents of BLS were higher than those in the control (*p* < 0.01). Moreover, the expression of chondrocyte/bone marker genes (collagen II, aggrecan, hyaluronan synthase 2, SOX 9), and osteogenic-specific genes (bone sialoprotein, osteocalcin, and osterix) was significantly upregulated in the BLS group (Natarajan et al., 2021).

Meanwhile, it is also important to consider the banded (zonal) structure of natural articular cartilage. Accordingly, Mancini et al. designed a scaffold with two layers of a thiol-ene cross-linkable HA/poly (glycidol) hybrid hydrogel [HA-SH/P (AGE-co-G)]. Articular cartilage progenitor cells (ACPC) and MSCs of superficial cartilage origin were added to the top and bottom layers, respectively. These layers were then mounted on 3D-printed poly (ε-caprolactone) (PCL) bone anchors, which were secured by reinforcing fibers protruding from the bone anchors onto the cartilage portion of the construct. Six months after implantation of the composite scaffold into an equine model, the mean compressive modulus of the repaired tissue in the banded group was 147.5 ± 40.7 kPa, which was significantly higher than that of the non-banded construct (96.9 ± 33.0 kPa, *p* < 0.05), however, lower than that of the natural cartilage (495.9 ± 174.0 kPa). Moreover, both the banded and non-banded groups formed fibrocartilage and produced primarily Col I rather than Col II and GAG, which may be related to the failure of MSC proliferation. At 6 months, both MSCs and ACPCs had disappeared leaving only host cells at the defect site, thus, fibrocartilage may be generated via host self-healing (Mancini et al., 2020). These findings may promote seed cell survival, adhesion, proliferation, and differentiation by culturing seed cells in a bioreactor or by adding appropriate growth factors.

Li et al. (2018b) designed a bionic three-layer fibrous-hydrogel scaffold and used a low-temperature 3D bioprinter to fabricate a three-dimensional bone and calcified layer scaffold with PLGA and β-TCP composite. The resulting product comprised a cartilage matrix from bovine articular cartilage using cell-free technology and freeze-drying technique. Li et al. (2018b) used the “lysis-adhesion technique” to fix the three layers together to obtain a stable three-layer bionic scaffold. MSCs were inoculated *in vitro*, and were found to adere to all scaffold layers. Moreover, the number of cells in the scaffold increased with time, and cell proliferation was stable during the plateau period from day 7–11. These results confirmed the biocompatibility of the scaffold, while the introduced calcified layer served as the role of bone-cartilage interface, achieving the expected isolation. However, considering the insufficient sample size and lack of animal model in this study, these findings require further investigation to demonstrate their feasibility and identify potential biological relationships (Li et al., 2018).

The upper interface between the CCZ and hyaline cartilage is called the tidemark, while the lower interface between the CCZ and subchondral bone is the cement line. In most studies of 3D printed regenerative osteochondral interfaces, little attention has been paid to the tidemark, which is difficult to induce after scaffold implantation. However, Mellor et al. (2020) and Nordberg et al. (2021) adopted the electrospinning technique to add a tidemark layer between the bone and cartilage layers. Their results demonstrated that the tidemark inhibits cell migration between the subchondral bone and cartilage, thereby preventing the invasion of cartilage by subchondral bone forming vessels (Mellor et al., 2020; Nordberg et al., 2021).

#### 3.4.4 Gradient Design of the Scaffolds

Osteochondral units are tissues that contain bone, cartilage, and transitional layers with gradient-based mechanical and biological properties. Therefore, a gradient-based scaffold design is essential for encapsulating the properties of musculoskeletal and other heterogeneous tissues (Bittner et al., 2018). Continuous gradient scaffolds more closely mimic natural OC tissue, as no distinct interface exists between each layer (Zhang et al., 2020b). However, in biphasic or multiphase interfaces the fixation between different interfaces may be unstable *in vivo,* thus, failing to mimic the original interface structure of osteochondral tissue, which is a gradual transition from soft cartilaginous tissue to hard subchondral bone, with biological, physicochemical, and anatomical gradients in the process.

Previous studies have suggested that parathyroid hormone (PTH) inhibits chondrocyte hypertrophy and facilitates articular hyaline chondrogenesis. Deng et al. (2021) used silk fibroin (SF) grafted with PTH by sulfonated SMCC (SF-PTH), covalently immobilized methacrylic anhydride (SF-MA), and photo-crosslinkable gelatin methacryloyl (GMA) for gradient strengthening of the scaffold based on natural mechanical strength. BMSCs were co-cultured separately *in vitro* with four bioinks (10% GM, 10% GM-5% SF, 10% GM-5% SF-MA, and 10% GM-5% SF-PTH). All four bioinks had good biocompatibility, while GM + SF-PTH ink inhibited the hypertrophy of culutred chondrocytes. After implantation of GM + SF-PTH/GM + SF-MA scaffolds in rabbit distal femoral talar sulcus defects, higher macroscopic scores, and fewer specific markers of chondrocyte hypertrophy, were observed compared with controls, demonstrating that this mechanically graded bioprinted biphasic scaffold can effectively promote regeneration of osteochondral defects, while PTH helps maintain the phenotype of hyaline cartilage (Deng et al., 2021).

Radhakrishnan et al. (2018) compared the effects of biphasic (nHA or CHS) and gradient (nHA + CHS) hydrogel scaffolds on *in vivo* osteochondral regeneration in a rabbit osteochondral defect model. The gradient group (8 weeks) had complete closure of the defect, showing good tissue coverage, while the other groups retained defects. Moreover, histological analysis revealed the formation of tidemark, collagen and GAG deposition in the neoplastic matrix, as well as the presence of hyaline cartilage, the characteristic matrix, chondrocytes, and osteoblasts. mCT further revealed mineralized new tissue formation and confinement to the defect area with a high bone density gradient (cartilage: 0.42 ± 0.07 g/cc, bone density: 0.64 ± 0.08 g/cc). In addition, biomechanical studies showed that the gradient group load for failure (378 ± 56 N) was significantly higher than that of the other groups. Thus, this bionic gradient hydrogel scaffold has the potential to promote osteochondral regeneration (Radhakrishnan et al., 2018).

Gao et al. (2019a) strengthened GelMA hydrogels by cleavable poly (N-acryloyl 2-glycine) (PACG) with dynamic hydrogen bonding and obtained hydrogels with high compressive strength (12.4 MPa) and compressive modulus (837 kPa). Moreover, bioactive glass (BG) can improve ALP activity, as well as the proliferation and differentiation of hBMSCs. Thus, the top layer of the generated hydrogel was doped with BG as the cartilage layer. Additionall, considering that Mn^2+^ can promote the cartilage differentiation of hBMSCs, the bottom layer of the hydrogel was doped with Mn^2+^ as the bone layer. The two layers were then fixed by UV light irradiation. The resulting bilayer biohybrid gradient hydrogel scaffold was evaluated using a rat model. The PAG-Mn-BG scaffold provided important template guidance and mechanical support, while further accelerating the regeneration of subchondral bone. Furthermore, the microscopic morphology of the repaired cartilage was smooth and homogeneous, with no significant difference from normal cartilage; that is, the scaffold enhanced both articular cartilage and subchondral bone repair and promoted the repair of osteochondral tissue at the defect site (Gao et al., 2019a).

In addition to biomechanical gradients, bioactive signal gradients are critical for regeneration at the osteochondral interface. SPIONs coupled with heparin produce a glycosylated corona that forms an agarose gel that stably encapsulates the BMP-2 gradient, which can effectively isolate and release growth factors. For example, a human HMSC stent pre-loaded with BMP-2 gradients released from the hydrogel over 28 days of culture, stimulated osteogenic gene expression and tissue mineralization. The resulting tissue exhibited a cartilaginous zone, rich in Col II and GAG, with transitioning to a mineralized bone zone exhibiting a broad distribution of β-TCP and HAP (Li et al., 2018a).

To date, many 3DP scaffolds have been fabricated to encapsulate the gradient properties of the bone-cartilage interface. Attempts have been made to mimic the chemical, mechanical, and biochemical structures, as well as the electrical gradients at the bone-cartilage interface. However, few scaffold materials with gradient metabolic properties have been developed. At the bone-cartilage interface, the vascularity is not distributed uniformly, thus, cells at different sites differ in terms of metabolic demand. Khorshidi and Karkhaneh (2021) designed a scaffold with oxygen-releasing particles from PLA and calcium peroxide. A gradient mixing chamber was employed to load the particles in a gradient manner into a hydrogel precursor solution of functionalized pectin and sericin. The chemical, morphological, and structural changes in the thickness of the composites were evaluated using microscopic and spectroscopic analyses. The particle concentration gradually increased from approximately 10% w/w over time and approached approximately 30% w/w by the end of the preparation process. SEM photographs of the composite cross-sections confirmed a gradual increase in the particle density from the lower surface to the upper surface. Meanwhile, spectral analysis confirmed that the scaffold was capable of releasing oxygen as a component, that is, calcium peroxide. Oxygen measurements of continuous cross-sections showed a gradual increase in oxygen production of the composite from the lowest to highest point. Microscopic and spectroscopic analyses confirmed the increase in particle content over the thickness of the scaffold. In addition, the scaffold cross-sections produced different amounts of oxygen and showed oxygen release behavior with depth (Khorshidi and Karkhaneh, 2021).

These innovative gradients are designed to promote hyaline cartilage formation by accelerating early subchondral bone regeneration and tight integration with the surrounding host site.

#### 3.4.5 Scaffold-Free Bioprinting

Stent implantation poses a myriad of problems, thus, the use of stentless bioprinting eliminates many of these complications while providing better intercellular interactions and long-term functions. Breathwaite et al. (2019) and Grogan et al. (2020) produced cellular microspheroids using MSCs to demonstrate the feasibility of cell-free scaffolds *in vitro* and in a rabbit osteochondral defect model, respectively. Meanwhile, Brown et al. (2021) used scaffold-free, self-assembling neocartilage as the chondral phase. They then compared the compressive strength of HAP, following introduction of HAp with 55% porosity with that of 0.95 MPa 32 at two neocartilage maturation stages (day 4 and 10). Osteochondral gross analysis, neocartilage and osteocondral histology, osteochondral interdigitation, neocartilage biochemistry, and neocartilage mechanics were then assessed. The early osteochondral assembly interface resulted in a 243-fold increase in shear modulus, a 4.9-fold increase in ultimate shear strength, a 244% increase in interface interdigitation depth, and a 438% increase in interdigitation frequency compared to late assembly (Brown et al., 2021).

### 3.5 Bioreactors

Even with the perfect combination of scaffolds, growth factors, and cells, osteochondral constructs may lack mass transfer of oxygen, nutrients, waste, and metabolites (Ravichandran et al., 2018). In *in vitro* cultures, cells are often loaded unevenly onto, and within, the scaffold, and cell viability and proliferation are heterogeneous throughout the graft. However, the flow state generated within the bioreactor helps overcome the limitations of oxygen diffusion in tissue-engineered grafts, while promoting cell transfer, providing critical physical and chemical cues for tissue regeneration, and helping restore the essential site properties of the original tissue, all of which is critical for maintaining cell survival and uniform cell distribution in the graft (Gadjanski, 2018). Biomechanical stimulation categories include direct compression, hydrostatic pressure, shear bioreactors, “low-shear” systems, and hybrid bioreactors that incorporate multiple loading regimes. Mechanical compression and shear forces represent the primary sources of physical stress affecting cartilage and subchondral bone.

**FIGURE 7:**
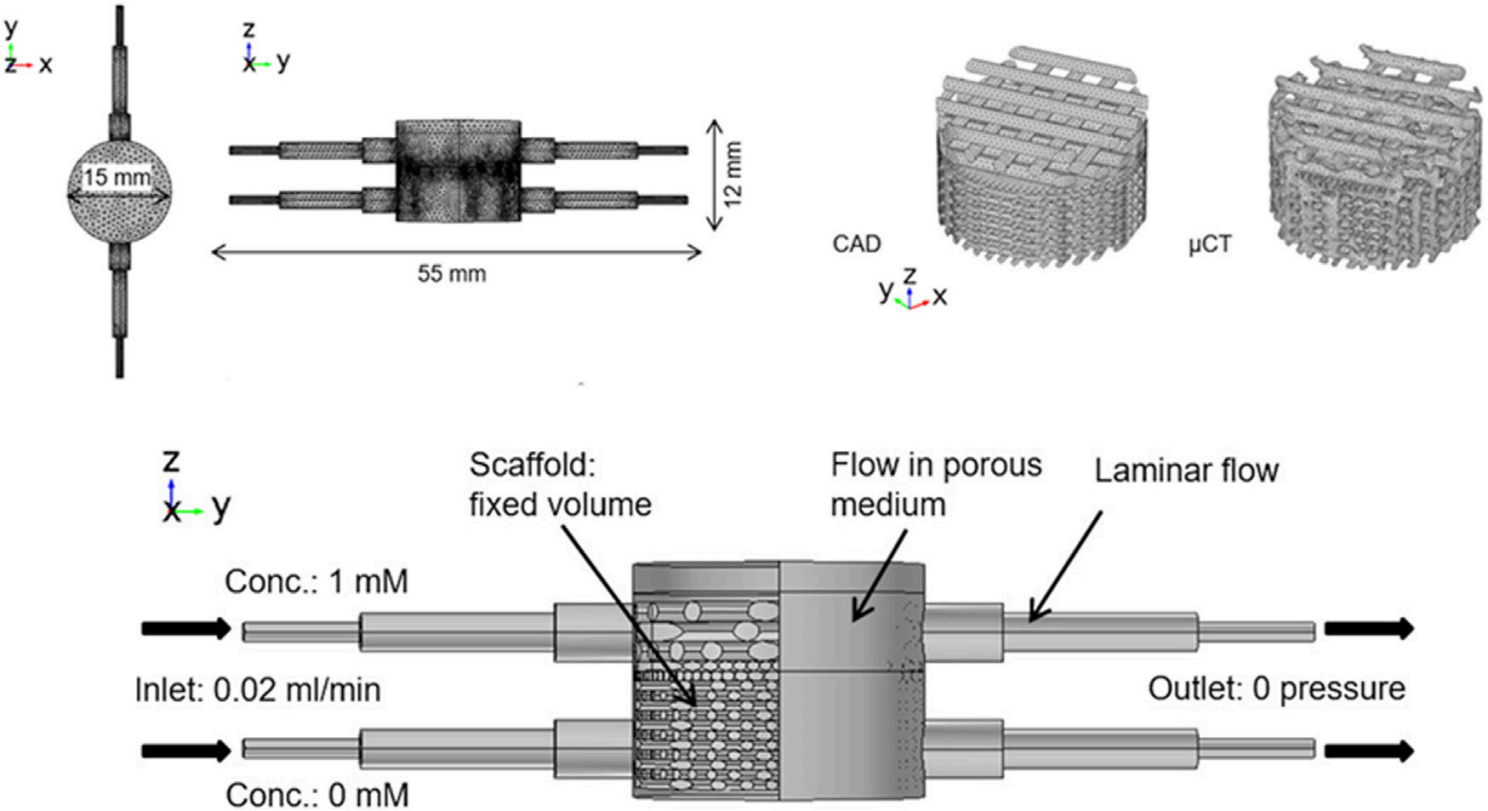
A representative structure of bioreactors. Reproduced with permission (Xue et al., 2019). Copyright: ^©^2019 by the authors.

Studies have shown that bioreactors (Figure 7) that can provide direct compression can stimulate chondrocytes and increase the synthesis of proteoglycans and collagen to enhance their mechanical properties. Fluid shear utilizes fluids to generate shear force between osteochondral constructs to increase the transfer of waste and nutrients during culture. Meanwhile, low-shear systems can be used to stimulate cells in the matrix, while still allowing the cells to retain their chondrocyte phenotype.

Data from computer modeling can be used to understand the correlation between physical stimuli and cellular responses to bone and cartilage formation, thereby, saving money and time required for *in vitro* and *in vivo* studies. Xue et al. (2019) proposed an osteochondral culture system using a flow rate of 0.02 ml/min and adding 1 (cartilage matrix) and 0 mM (osteogenic medium) concentration with no pressure at the outlet, inducing an average fluid-induced shear stress of approximately 0.03 and 0.28 MPa in the cartilage and bone layers. Bilayer PLA scaffolds loaded with ATDC5 and MC3T3-E1 cells cultured in this system for 7 days showed a significant increase in metabolic activity and cell number compared to CAD scaffolds. ATDC5 cells dominated the upper segment, while MC3T3-E1 cells dominated the lower segment. Moreover, the cells effectively attached to the collagen and PLA struts of the scaffold, thus, demonstrating the viability of the system (Xue et al., 2019). However, this system is not only applicable to *in vitro* culture of osteochondral bone, but also for the cytotoxicity and response monitoring of clinical drugs.

Nichols et al. optimized a new bioreactor capable of optical monitoring within 3D structures (Nichols et al., 2018). Yu et al. (2020) designed and developed a polydimethylsiloxane coculture system for osteochondral tissue (PCSOT). The body of PCSOT consists of a PCL membrane divided into two separate chambers, allowing cells to be exposed to different culture media using different chondrogenic and osteogenic media, thereby, providing a suitable osteochondral differentiation environment that allows FCPC to differentiate into osteochondral tissue (Yu et al., 2020). Current research is focused on unifying the design of bioreactors for effective osteochondral graft culture.

## 4 Discussion and Future Directions

With the development of 3DP technology and a deeper understanding of osteochondral structure, researchers began to consider the feasibility of applying 3DP to regenerate OCD, from simply repairing articular cartilage to subchondral bone and smooth bone-cartilage interfaces. Initially, natural materials were the first choice for constructing scaffolds; however, their disadvantages could not be avoided. Currently, composites based on natural and artificial polymers are the dominant research directions. Due to the location of the bone-cartilage interface, regenerating the bone-cartilage interface must consider both articular cartilage and subchondral bone. Although the approach based on monophasic scaffolds has become obsolete, many research groups have developed bilayer and triple-layer scaffolds that mimic the osteochondral cartilage and bone layering structure (or tidemark and calcified cartilage area). These scaffolds have also been combined with tissue-specific cells (osteoblasts for bone, chondrocytes for cartilage) or MSCs (BMSCs, hTMSCs, AMSCs, and UCB-MSCs) and appropriate growth factors are then selected to promote migration, proliferation, and differentiation of the seed cells to form osteochondral tissue. The development of mechanical gradient scaffolds with a structure mimicking osteochondral tissue and bio-gradient scaffolds with graded release of bioactive factors is promising for establishing the formation of osteochondral interfaces. Bioreactor culture further facilitates homogeneous nutrient transfer, providing key physical and chemical cues for tissue regeneration and promoting osteochondral tissue formation. 3D scanning (Li et al., 2017) and robot-assisted 3D bioprinting (Lipskas et al., 2019; Ma et al., 2020) are viable options. In the last 5 years, many combinations have been proposed and have been successful. However, some key challenges remain, including how to differentiate different tissues (bone and cartilage) while regenerating the bone-cartilage interface so that the regenerated tissue structurally and functionally mimics the native tissue. Animal models are also vital for clinical translation. Although small animals like rats and rabbits have the advantages of lost cost, but for the consideration of defect size and surgical difficulty, researchers should focus more on the big animal models for the evaluation of 3D printing Scaffolds’ clinical prospects (Table 1). In addition, clinical applications face many regulatory and commercial challenges while needing to accommodate the automation and volume of composite scaffold printing.

**TABLE 1:** Current clinical translational results of 3D printing for OCD regeneration in big animal models.

Materials	Cell/molecules type	Scaffold structure	Development	Result	References
RGD-γ alginates, PCL	FPSCs, chondrocytes, BMSCs	Bi-phasic	In caprine models	After 6 months of implantation, osteochondral tissues were generated significantly. However, limited Safranin-O staining suggested the cartilage template have undergone endochondral ossification. One animal’s implantation failed	Critchley et al. (2020)
—	AT-MSC	Scaffold free	In mini-pig models	After 3 months of operation, percentage RV and MOCART scores had significant differences compared with the control group. After 6 months of operation, the gross scores were higher than the control group but without statistical differences	Yamasaki et al. (2019)
HA-SH/P (AGE-co-G), PCL	ACPCs, MSCs	Tri-phasic	In equine models	Observed promising results of bone regeneration in equine models. However, the cartilage regeneration was worse than the natural OCD groups	Mancini et al. (2020)
PCL, collagen type I gel	hASC	Tri-phasic	In porcine models	The scaffold reinforced with intermediate electrospun layer had better performance and operational convenience than the single PCL scaffold	Mellor et al. (2017)
PCL, TCP, dECM	hASC	Bi-phasic	In porcine models	The scaffolds promoted the regeneration of osteochondral tissues compared with the open lesion groups. Scaffold loaded with hASC scored best ICR II grading among all groups. Adding a tidemark layer performed as a boundary line to separate the cartilage and bone	Nordberg et al. (2021)

GelMA, gelatin methacrylate; ECM, extracellular matrix; MSC, mesenchymal stem cell; PCL, polycaprolactone; FPSCs, fat pad derived stem/stromal cells; BMSCs, bone marrow derived stem cells; AT-MSC, adipose tissue-derived mesenchymal stem cells; ACPC, articular cartilage progenitor cells; HA-SH/P(AGE-co-G), thiol-ene cross-linkable hyaluronic acid/poly(glycidol) hybrid hydrogel; hASC, human adipose-derived stem cells; TCP, β-tricalcium phosphate.
